# 

**Published:** 1918-01-12

**Authors:** 


**Rates of Subscription:** Twelve months, 6/6; Six months, 4/-; three months, 2/-, post free to any address in the United Kingdom. Abroad, Twelve months, 8/8; Six months, 5/-; Three months, 2/6, post free. THE HOSPITAL "Index" to Volume LXII., April 1917 to September 1917, is now ready, price 2½d. per copy, post free. Address The Manager, The Hospital, "The Hospital" Building, 28/29 Southampton Street, Strand, London, W.C. 2.

### HOSPITAL AND INSTITUTIONAL RESULTS.

**Royal Dental Hospital of London.**—Although the War Office has taken over a large portion of the hospital, the number of patients treated in 1916 was 17,296, and their attendances 37,239. The corresponding numbers in the previous year were 25,834 and 70,855. Regular soldiers of the Active Army are now treated by the War Office instead of by the hospital. A surplus of £1,193 on the year's working has been used for repayment on account of the mortgage, which now stands at £27,109.

**Royal Southern Hospital, Liverpool.** — The annual report contains 129 pages, an index, several insets, and a number of illustrations. The cost per occupied bed has increased from £77 0s. 3d. in 1915 to £85 2s. 2d. in 1916. As the waiting list of civil patients at the beginning of the year had been greatly reduced, twenty additional beds were allotted to sick and wounded soldiers. The Liverpool Insurance Committee pays the hospital per week 27s. 6d. for adults and 12s. 6d. for children suffering from tuberculosis. Over £9,000 is owing to the bankers.

**Pontypool and District Hospital.**—This institution issues a half-yearly report and financial statement. Up to June 30, £2,791 had been collected, all ordinary expenses had been paid, and, after £992 had been transferred to the building fund, there remained £290 in hand. The workmen of the neighbourhood contributed £1,680, and the employers £374. The Garndiffaith Workmen's Institute has subscribed £500 to provide a motor ambulance.

**Royal Albert Edward Infirmary and Dispensary, Wigan.**—This institution, for some probably forgotten reason, has a financial year ending on March 31. Now is the time to alter this, when the world is at sixes and sevens. The same opportunity might be taken to arrange the income and expenditure accounts on the Uniform System.

**West Bromwich and District Hospital.**—Thirteen pages of the report are filled by the names of committees and officers, including those belonging to the Hospital Saturday Committees. Not only money gifts, but gifts in kind, are acknowledged in detail. The report is tastefully printed and well arranged; the only fault we can find is that the financial year is made to end on June 30. Why not December 31, in accordance with the practice of the great majority of institutions?

**Worcester General Infirmary.**—There appears to have been less call on the resources of the hospital in 1916 than in 1915; there was a 20 per cent. decrease in the number of in-patients, and the out-patients were also fewer. We notice on page 7 that a gift of £800, part of a discretionary bequest, was received through the Public Trustee, but this cannot have been included under donations, as the total of that item was only £745. Has the amount, contrary to the latest approved practice, been entered as a legacy, or where is it entered?

**Orphan Working School.**—This charity has its own way of presenting its accounts, and this way is not a particularly good way. For instance, a sum of £4,078 is stated to have been spent during 1916 on "housekeeping." What this word is intended to describe is not clear. Is it food? If so, why not say so; in short, why not arrange the accounts in the form adopted by most other charities, which would give the subscribers and the public an opportunity of forming an opinion as to economical management?

**Wolverhampton and Staffordshire General Hospital.**—The annual report contains 111 pages and three plans printed on good thick paper. The hospital is liberally supported; no legacies are included in the income and expenditure account, and yet there was a surplus during 1916 of £3,006.

### Matrons' and Sisters' Appointments.

Charing Cross Hospital.—Miss Mary Cochrane has been appointed night sister at this hospital, where she was trained.

County Hospital, York.—Miss M. W. West has been appointed theatre sister. She was trained at the Royal Infirmary, Sheffield, and at the Children's Hospital, Western Bank, Sheffield.

East London Hospital for Children, Shadwell.—Miss Judith Hancock has been appointed assistant matron and home sister. She was trained at the Children's Hospital, Sheffield, Seamen's Hospital, Greenwich, and Samaritan Hospital for Women, and has been sister at the Belgrave Hospital for Children, the Samaritan Hospital for Women, and at the East London Hospital for Children. She was for three years a member of the Royal Naval Nursing Service Reserve.

Hartlepools Hospital, Hartlepool.—Miss Pauline Holden has been appointed night sister. She was trained at the District Infirmary, Ashton-under-Lyne, and has since been staff nurse at the Southport Infirmary.

Hospital for Paralysed Sailors and Soldiers, Nottingham.—Miss M. R. Ward has been appointed matron. She was trained at St. Thomas's Hospital, and has since been matron at the Cottage Hospital, Bushey Heath, Watford.

Ulster Hospital for Children and Women, Belfast.—Miss Margaret H. Cherry has been appointed sister. She was trained at the Royal Victoria Hospital, Belfast, afterwards becoming holiday sister there.

Weston-super-Mare Hospital.—Miss Ruth Heatley has been appointed matron. She was trained at Manchester Royal Infirmary, has been sister at the Children's Hospital, Coldash, Newbury, and at the Harrow Cottage Hospital, and matron at Fleet Cottage Hospital. She has also been doing military work in France.

**NOTE.—Particulars of Appointments for insertion in this column will be welcomed.**

### King Edward's Hospital Fund for London.

A meeting of the General Council of King Edward's Hospital Fund for London was held on January 7, at the offices of the Fund, 7 Walbrook, E.C. 4. Lord Somerleyton (honorary secretary) stated that the Governors had appointed the following new members of the General Council: The Viscount Astor, Mr. Yvon R. Eccles, and Mr. Geo. Lawson Johnston. The order of appointment, signed by the Governors, of the members of the General Council, members of Committees, and officers for the year 1918 was read.

The resolutions providing for the work of the Fund for 1918, which were approved at the meeting of the Governors and General Council held at St. James's Palace on December 13, 1917, were formally adopted.

